# Pathophysiology of ocular surface squamous neoplasia

**DOI:** 10.1016/j.exer.2014.10.015

**Published:** 2014-12

**Authors:** Stephen Gichuhi, Shin-ichi Ohnuma, Mandeep S. Sagoo, Matthew J. Burton

**Affiliations:** a London School of Hygiene and Tropical Medicine, Keppel Street, London WC1E 7HT, UK; b Department of Ophthalmology, University of Nairobi, P.O Box 19676-00202, Nairobi, Kenya; c UCL Institute of Ophthalmology, 11-43 Bath Street, London EC1V 9EL, UK; d Moorfields Eye Hospital, 162 City Road, London EC1V 2PD, UK; e St. Bartholomew's Hospital, W Smithfield, London EC1A 7BE, UK

**Keywords:** Pathophysiology, Ocular surface squamous neoplasia (OSSN), Limbal stem cells, Cancer stem cells, Ultraviolet radiation, p53, HPV, HIV

## Abstract

The incidence of ocular surface squamous neoplasia (OSSN) is strongly associated with solar ultraviolet (UV) radiation, HIV and human papilloma virus (HPV). Africa has the highest incidence rates in the world. Most lesions occur at the limbus within the interpalpebral fissure particularly the nasal sector. The nasal limbus receives the highest intensity of sunlight. Limbal epithelial crypts are concentrated nasally and contain niches of limbal epithelial stem cells in the basal layer. It is possible that these are the progenitor cells in OSSN. OSSN arises in the basal epithelial cells spreading towards the surface which resembles the movement of corneo-limbal stem cell progeny before it later invades through the basement membrane below. UV radiation damages DNA producing pyrimidine dimers in the DNA chain. Specific CC → TT base pair dimer transformations of the p53 tumour-suppressor gene occur in OSSN allowing cells with damaged DNA past the G1-S cell cycle checkpoint. UV radiation also causes local and systemic photoimmunosuppression and reactivates latent viruses such as HPV. The E7 proteins of HPV promote proliferation of infected epithelial cells via the retinoblastoma gene while E6 proteins prevent the p53 tumour suppressor gene from effecting cell-cycle arrest of DNA-damaged and infected cells. Immunosuppression from UV radiation, HIV and vitamin A deficiency impairs tumour immune surveillance allowing survival of aberrant cells. Tumour growth and metastases are enhanced by; telomerase reactivation which increases the number of cell divisions a cell can undergo; vascular endothelial growth factor for angiogenesis and matrix metalloproteinases (MMPs) that destroy the intercellular matrix between cells. Despite these potential triggers, the disease is usually unilateral. It is unclear how HPV reaches the conjunctiva.

## Highlights

• Limbal epithelial stem cells are the likely progenitor cells of OSSN.
• UV radiation causes DNA damage via dimers, photoimmunosuppression and reactivates latent HPV.
• HPV E6 inhibits p53 tumour suppressor gene allowing DNA-damaged cells past the G1-S checkpoint of the cell cycle.
• HPV E7 inhibits pRB gene antitranscription at G1 so infected cells keep replicating.
• HIV, photoimmunosuppression and vitamin A deficiency impair tumour surveillance.

## 1 Introduction

Ocular surface squamous neoplasia (OSSN) comprises of a spectrum of tumours that affect the ocular surface ranging histologically from intraepithelial neoplasia to different grades of invasive squamous cell carcinoma (Lee and Hirst, 1995). Early lesions of varying size usually occur at the limbus, the area of transition between the cornea and conjunctiva (Lee and Hirst, 1997, Waddell et al., 2006). Advanced stages may involve the eyelids and may invade the orbit. Curiously OSSN usually affects only one eye (Chisi et al., 2006).

OSSN occurs worldwide but the peak incidence is found at a latitude of 16° South (Gichuhi et al., 2013). The mean age-standardised incidence rate worldwide is 0.18 and 0.08 cases/year/100,000 among males and females, respectively and the highest incidence rate is found in Africa (1.38 and 1.18 cases/year/100,000 in males and females) (Gichuhi et al., 2013). In temperate countries OSSN predominantly affects males while in Africa both sexes are affected equally. Systematic reviews and meta-analysis show that the main risk factors are solar ultraviolet (UV) radiation, HIV and human papilloma virus (HPV); while vitamin A deficiency is a potential risk factor but has not been investigated (Gichuhi et al., 2013, Carreira et al., 2013). This paper reviews the pathophysiological mechanisms underlying the development of OSSN.

## 2 Ocular surface anatomy

The ocular surface consists of the cornea, limbus and conjunctiva but in a wider anatomical and embryological sense the mucosa of the ocular adnexa (lacrimal gland and lacrimal drainage system) is included. The epithelia of the cornea, conjunctiva and eyelid are formed from differentiation of the surface ectoderm during embryonic development. The corneal endothelium and the corneal stroma, conjunctiva and eyelids are formed when periocular mesenchymal cells of neural crest origin migrate and differentiate (Cvekl and Tamm, 2004, Kao et al., 2008).

The cornea has a stratified squamous non-keratinizing epithelium with five to seven cell layers. It is immunologically privileged due to its lack of blood vessels and lymphatics, with dendritic cells present usually only in the peripheral cornea (Akpek and Gottsch, 2003).

**Fig. 1:**
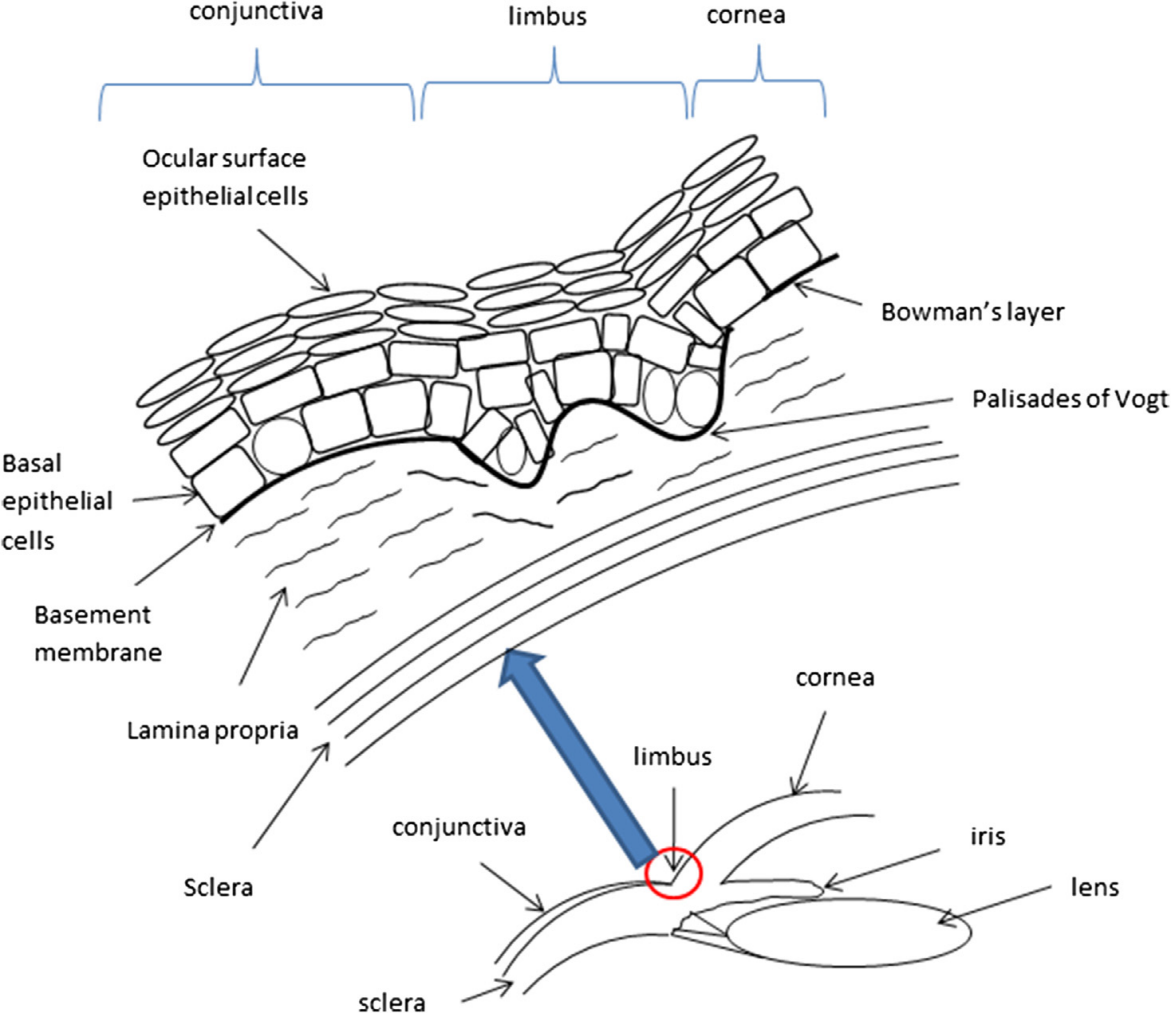
Anatomy of the limbus.

The limbal epithelium is 8–10 cells thick and is constantly being replenished from stem cells in the basal layer (Schermer et al., 1986). The limbal basement membrane has undulating peg-like inter-digitations into the underlying stroma called the palisades of Vogt, which increase the surface area and protect against shearing forces (Fig. 1). The palisades are unique for individuals (like fingerprints) and have distinct radial vascular loops that leak fluorescein in the late phase of angiography suggesting a protective function for stem cells (Goldberg and Bron, 1982). The basal cells are protected from UV light by melanin within deep limbal crypts, where melanocytes contain melanin granules oriented towards the apex of each cell, acting as a pigmented cap facing the ocular surface (Higa et al., 2005). Among darker pigmented races the limbus is heavily pigmented, perhaps offering greater protection from UV radiation.

The conjunctiva consists of an epithelium on a basement membrane and underlying loose connective tissue called the lamina propria. The lamina propria is loosely anchored to the episclera and sclera making the conjunctiva easily mobile. The epithelium varies between 2–3 and 10–12 cell layers, depending on whether it is the bulbar, fornix or tarsal portion. Lymphocytes and plasma cells are abundant in the conjunctiva (Hingorani et al., 1997). They form the conjunctiva-associated lymphoid tissue (CALT) in the lamina propria (Knop and Knop, 2007).

## 3 Limbal stem cell biology

Stem cell biology is a rapidly progressing field. A stem cell is a special undifferentiated progenitor cell capable of giving rise to many more cells of the same type, and from which other kinds of cells arise by differentiation. There are three types of stem cells. Embryonic stem cells originate from pre-implantation embryos and can develop into tissues that belong to one of the three germ layers (Martin, 1981). Non-embryonic adult stem cells (termed somatic) are undifferentiated cells found in special niches of various organs where they divide and differentiate to replace damaged tissue while some may transdifferentiate to other tissues (Gonzalez and Bernad, 2012). Their origin remains unclear. Limbal epithelial cells would fall in this category. Lastly, induced pluripotent stem cells are created in the lab by genetically reprogramming somatic cells to an embryonic stem cell-like state (Takahashi et al., 2007, Obokata et al., 2014).

Corneo-limbal lineage is distinct from conjunctival lineage (Wei et al., 1996). Evidence suggests the existence of corresponding stem cell reservoirs. Corneo-limbal epithelial stem cells are located in the limbal basal layer while conjunctival stem cells are distributed throughout the bulbar and forniceal conjunctiva, but some propose that they are concentrated in the fornix (Nagasaki and Zhao, 2005, Sun et al., 2010, Pellegrini et al., 1999, Wei et al., 1995). Stem cells are also found in the corneal stroma and mucocutaneous junction of the lid margin (Du et al., 2005, Wirtschafter et al., 1997). The molecular structure of the basement membrane and extracellular matrix at the cornea, limbus and conjunctiva differ from each other and this is thought to play a role in regulation of epithelial differentiation (Kolega et al., 1989, Schlotzer-Schrehardt et al., 2007). Various markers have been studied in the ocular surface to determine which are more concentrated at the limbus (Table 1) (Schlotzer-Schrehardt and Kruse, 2005). There are no specific limbal stem cell markers or any that distinguishes stem cells from their early progeny (Chee et al., 2006).

Limbal stem cells are found in special niches in the basal region called limbal epithelial crypts which are cords of epithelial cells extending from the palisades of Vogt into the underlying stroma (Dua et al., 2005). The crypts are most abundant nasally in the mid- or distal limbus (Shanmuganathan et al., 2007). Stem cells within the crypts have the following marker profile; CK3−/CK19+/CD34−/Vimentin+/p63+/Connexin43+/Ki67− (Shanmuganathan et al., 2007). Stem cells represent less than 10% of the limbal basal cell population (Lavker et al., 1991). They are characterised by; low level of differentiation, slow cell-cycle, long life-span, high proliferative potential and self-renewal (ability to produce more stem cells) (Schlotzer-Schrehardt and Kruse, 2005, Dua and Azuara-Blanco, 2000). The limbus creates a barrier to prevent extension of conjunctival epithelium and blood vessels into the cornea (Osei-Bempong et al., 2013). Clinical features of limbal stem cell deficiency disorders thus include corneal epithelial defects, conjunctival epithelial migration onto the cornea and corneal neovascularization (Sejpal et al., 2013).

The ocular surface is self-renewing. Superficial cells are constantly lost and are replaced by basal cells entering the differentiation pathway. To replenish the corneal epithelium, corneal epithelial stem cells undergo mitosis producing a progeny of fast-dividing transient amplifying cells (TAC) that make up the majority of the proliferating cell population in the epithelium (Castro-Munozledo, 2013). TAC migrate superficially to the suprabasal limbus and centripetally towards the centre of the cornea to form the basal layer of the corneal epithelium (Lehrer et al., 1998). They undergo a limited number of divisions then differentiate into post-mitotic cells (PMC) and further into terminally differentiated cells (TDC) that migrate superficially to the corneal surface (Lehrer et al., 1998). It takes 14–21 days for complete renewal of the rabbit corneal epithelium (Haddad, 2000). In humans it takes 5–7 days (Hanna and O'Brien, 1960). For conjunctival renewal, cells stream centrifugally (instead of centripetally as occurs in the cornea) at 10.5 ± 2.4 μm/day then superficially at 9.3 ± 5.4 μm/day with a cell-cycle time of 8.3 days in rats (Zajicek et al., 1995). Stem cells have a slower passage through the cell cycle than the other basal cells of the cornea and conjunctiva (Lavker et al., 1991). In mice they take 4–8 weeks and are preferentially stimulated by wounding and tumour promoting compounds (Cotsarelis et al., 1989). Intact innervation of the ocular surface is needed to maintain the stem cell niche (Ueno et al., 2012).

## 4 Cancer stem cells

The term cancer stem cell is a relatively new one in cancer biology, though this is a concept known for many years (Wicha et al., 2006). In malignant tumours there is frequently a subpopulation of cells that responds poorly to treatment such as chemotherapy and radiotherapy, divides at a slower rate than other cancer cells, and is less affected by hypoxia (Moore and Lyle, 2011, Lin and Yun, 2010). This subpopulation is thought to drive tumour growth and is the subject of much debate and much investigation for different tumour types. The origin of these cells is controversial and their interaction with non-stem cancer cells has been variously studied using mathematical models (Wang et al., 2014).

Cancer stem cells comprise less than 5% of the cell population in most tumours (Yang and Chang, 2008). They are found in various cancers including breast, brain, gastric, pancreatic and liver (Al-Hajj et al., 2003, Yuan et al., 2004, Takaishi et al., 2008, Lee et al., 2008, Sell and Leffert, 2008). In high-grade cervical intraepithelial neoplasia (CIN) associated with carcinogenic HPV types they are found at the squamo-columnar junction but rarely in ectocervical/transformation zone CINs or those associated with non-carcinogenic HPVs (Herfs et al., 2012).

Their existence could help to explain the clinical observation of the inaction of conventional cancer chemotherapy and radiotherapy in some instances since these treatments target rapidly dividing cells yet stem cells by nature are slow-cycling. For example, treatment with fluorouracil (5-FU) selects and enriches the cancer stem cell population since the rapidly dividing cells would be killed while slow-cycling ones incorporate the drug at a lower rate (Shi et al., 2013, Wang et al., 2006). Potentially, therapies targeting cancer stem cells could be more effective (Duggal et al., 2014).

## 5 Patterns of ocular surface dysplastic and neoplastic disease

**Fig. 2:**
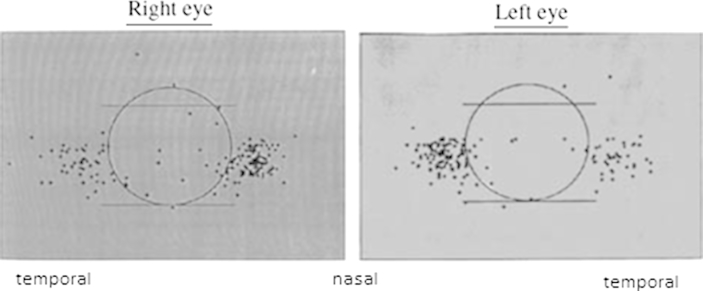
Location of 352 OSSN tumours in Uganda showing most lesions occurred within the interpalpebral fissure with a higher concentration in the nasal sector.

**Fig. 3:**
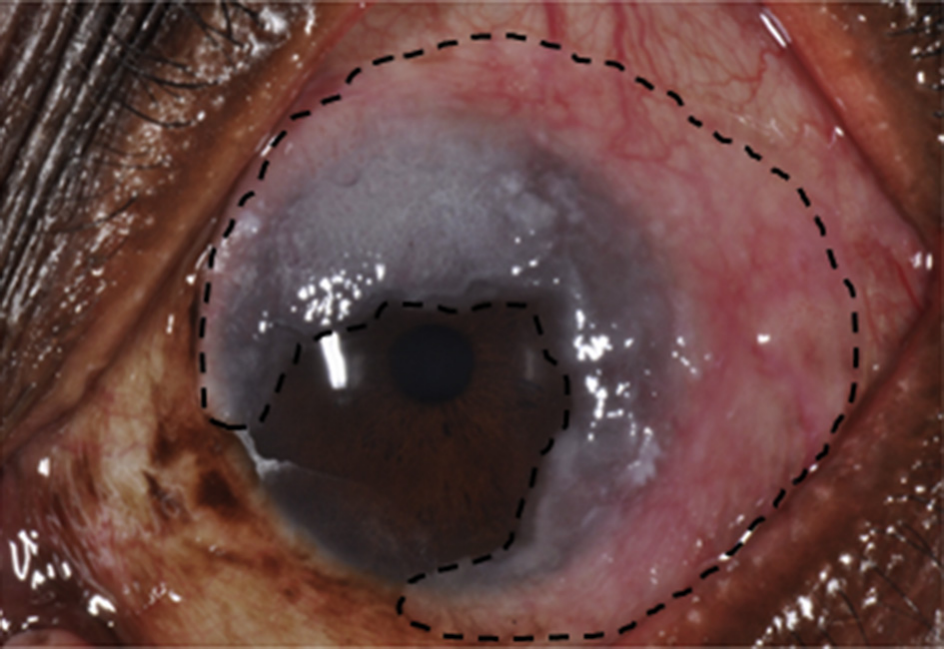
A lesion of OSSN lesion in Kenya showing a circum-limbal growth pattern involving almost the entire circumference of the limbus. The margins are drawn in a black dotted line to show extension into the cornea and bulbar conjunctiva.

The clinical presentation of ocular surface dysplastic and neoplastic diseases provides clues to the pathophysiology of OSSN. Firstly, OSSN, pterygium, pingueculae, climatic droplet keratopathy and actinic keratosis are usually located within the interpalpebral fissure, the space between the open upper and lower eyelid that is exposed to UV radiation (Waddell et al., 2006, Sudhalkar, 2012, Shields et al., 2004, Gray et al., 1992). Secondly, most OSSN lesions arise from the limbus particularly the nasal quadrant (Fig. 2) (Waddell et al., 2006). A similar observation was made of pterygia in India where all the lesions in a study of 427 participants were nasal (Sudhalkar, 2012). This is the area with the highest concentration of limbal epithelial crypts (Shanmuganathan et al., 2007). Thirdly, the disease may involve the circumferential limbus with relatively little involvement of the cornea or fornix (Fig. 3). Lastly, intraepithelial neoplasia begins in the basal cells and spreads upwards, a pattern reflected in the histological grading (Basti and Macsai, 2003, American Joint Committee On Cancer, 2010). This resembles the spreading waves of limbal stem cells and their progeny described in biology above suggesting that the disease may be of stem cell origin. It remains unclear why OSSN is often unilateral since exposure to UV radiation, HIV and HPV has no laterality.

## 6 Vulnerability of the limbus

**Fig. 4:**
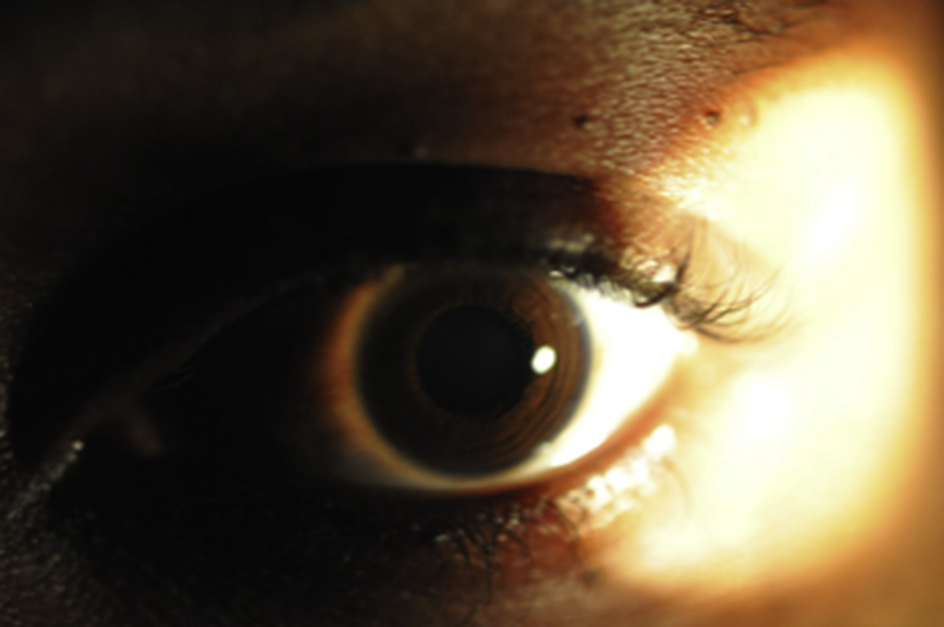
Light from a torch shining on the temporal side of the eye to illustrate that the limbus receives direct sunlight temporally which is focused nasally. Notice the glow in the nasal limbus.

The limbus receives direct sunlight temporally which is focused nasally (Fig. 4). As the human eye is more exposed laterally, the large temporal visual field becomes a collecting zone of peripheral light, which, depending on the angle of incidence (*θ*) and the corneal central radius of curvature (*r*_0_), is intensely focused onto the nasal limbus, lid margin or lens with up to a 20-fold increase in peak intensity (Maloof et al., 1994). Temporal light that traverses the anterior chamber strikes the nasal limbal cells basally where there is less melanin. These foci coincide with the usual site for pterygium, OSSN, lid malignancy and cataract.

Limbal basal cells remain quiescent but proceed more rapidly through the cell cycle when there is an insult to the ocular surface such as a wound or tumour promoter (Cotsarelis et al., 1989). Quiescent adult stem cells accumulate DNA damage making them vulnerable to neoplastic transformation (Mohrin et al., 2010). Others however suggest that slow-cycling protects them from cancer (Wodarz, 2007). Quiescence may create a reservoir of latent virus infection, which can persist for long periods before reactivation following immunosuppression (Maglennon et al., 2011). In skin hair follicle stem cells the papilloma virus oncogenes, E6 and E7, can compromise stem cell quiescence by promoting their aberrant mobilization (Michael et al., 2013).

## 7 Key events in the aetiology of OSSN

### 7.1 DNA damage: genetic and epigenetic changes

At the heart of carcinogenesis is non-lethal DNA damage. DNA damage can be genetic (mutations of the DNA nucleotide sequence) or epigenetic (variations in gene expression that do not involve changing the nucleotide sequence). DNA damage affects genome stability and stem cell function leading to cancer (Xu and Taylor, 2014). Mutations may affect oncogenes (genes that facilitate cell division) or tumour suppressor genes (that slow down or stop cell division). Epigenetic modifications can take three different forms (Walters, 2013). Firstly, DNA methylation where cytosine nucleotides (C) are found adjacent to guanine nucleotides (G) called CpG sites, which shuts down RNA transcription. Secondly, modifying histones (e.g. acetylation and methylation) around which DNA is wrapped allowing uncontrolled access to DNA by transcription factors. Thirdly, silencing micro RNA (miRNA) genes which regulate cell processes such as proliferation, differentiation, and apoptosis by binding the 3′ untranslated region of target mRNA. Epigenetic changes are often reversible (Delcuve et al., 2009). Although distinct from each other, epigenetic changes and mutations are related because epigenetic changes may lead to mutations and cancer (Feinberg et al., 2006). Ocular surface DNA damage is probably mainly caused by solar UV radiation (UVR), although HPV may also play a role.

#### 7.1.1 Effects of solar ultraviolet radiation

##### 7.1.1.1 Genetic and epigenetic changes

Ambient UVR can be broadly divided into UVA (320–400 nm, approximately 90%) and UVB (290–320 nm, approximately 5%) wavebands (Diffey, 2002). UVC (200–290 nm) is largely prevented from reaching the earth's surface by the ozone layer in the atmosphere.

UVB radiation causes direct DNA damage by crosslinking adjacent bases to form cyclobutane pyrimidine dimers (CPDs) and 6-4 photoproducts (6-4 PPs) (Pfeifer et al., 2005). The most commonly seen are CPDs and are considered the hallmarks of UV damage (Besaratinia et al., 2011). Pyrimidine dimers are formed when adjacent bases (thymine-T or cytosine-C) on the same DNA strand absorb energy from UV light and form crosslinks via carbon-to-carbon covalent bonds (Rastogi et al., 2010). CPDs and 6-4PPs distort DNA's structure and block DNA synthesis by preventing the replicative DNA polymerases from moving along a template strand (Ikehata and Ono, 2011). CPDs are also resistant to hydrolysis (Yamamoto et al., 2014). Specific CC → TT dimer transitions of the p53 tumour-suppressor gene have been observed in OSSN lesions in Uganda (Ateenyi-Agaba et al., 2004). p53 mutations occur in different phases of the multistep malignant transformation and can be found in precancerous lesions such as actinic keratosis (Rivlin et al., 2011). The effects of p53 mutation are discussed later in this article.

UVA causes indirect DNA damage via reactive oxygen species (ROS), like ^−^OH (hydroxyl radical), O_2_^−^ (superoxide radical anion) or H_2_O_2_ (hydrogen peroxide) leading to DNA strand breaks (Cortat et al., 2013, Greinert et al., 2012, Cadet et al., 2009). No studies have demonstrated DNA strand breaks in OSSN. Cells have greater ability to repair UVA effects than UVB (Besaratinia et al., 2008).

##### 7.1.1.2 Reactivation of latent HPV infection

Exposure to UVB reactivates latent HPV(Zhang et al., 1999), HIV(Breuer-Mcham et al., 2001), varicella-zoster (shingles) (Zak-Prelich et al., 2002, Rice, 2011) and herpes simplex (cold sores) (Blatt et al., 1993, Miller et al., 1993). HPV is a small DNA virus. Asymptomatic HPV infection is widespread with an estimated global prevalence of 11.7% in women and 1.3%–72.9% in men (Bruni et al., 2010, Dunne et al., 2006). HPV is epitheliotropic for squamous epithelia especially transitional mucosae and is implicated in the aetiology of various squamous cell carcinomas including cervical (summary OR = 70.0, 95% CI; 57.0–88.0), colorectal (summary OR = 10.0, 95% CI; 3.7–27.5), laryngeal (summary OR = 5.4, 95% CI; 3.3–8.9), OSSN (summary OR = 4.0, 95% CI; 2.1–7.6), oesophageal (summary OR = 3.3, 95% CI; 2.3–4.9) and bladder (summary OR = 2.8, 95% CI; 1.4–5.8) (Munoz, 2000, Damin et al., 2013, Li et al., 2013b, Li et al., 2014, Li et al., 2011, Gichuhi et al., 2013, Li et al., 2014, Li et al., 2011).

Much of what is known about the pathophysiology of HPV in cancer is derived from cervical studies. HPV invades the basement membrane through micro abrasions where it initially binds to heparin sulphate proteoglycan (HSPG) (Johnson et al., 2009, Sapp and Bienkowska-Haba, 2009). It does not bind intact epithelia (Johnson et al., 2009). The basement membrane is not merely a passive reservoir of virus but is involved in viral processing. Here the viral capsids undergo a conformational change where the L2 epitope is cleaved by a protease and exposure of its N-terminal leads to the transfer of capsids to the epithelial cell surface (Kines et al., 2009). After internalization, the virus is disassembled and the DNA enters the nucleus by a mechanism that is still not well understood where it replicates producing extra-chromosomal copies of viral DNA (Sapp and Bienkowska-Haba, 2009). However the genome of high-risk HPV has been found incorporated into specific preferential sites of the host DNA in cervical lesions (Li et al., 2013a). During differentiation of epithelial cells, virions mature and are carried towards the surface (Doorbar, 2005). In normal uninfected epithelia, as cells leave the basal layer, they exit the cell cycle but infected cells remain active due to E7 (Longworth and Laimins, 2004). E7 inactivates the retinoblastoma gene (pRB) which usually acts in the G1 phase of the cell cycle where it binds transcription factors, thus infected cells remain in a proliferative state while E6 binds the p53 protein preventing it from suppressing replication of such DNA-defective cells (Lehoux et al., 2009). In high-risk HPV types, E6 and E7 also cause genomic instability; E7 causes centriole over-duplication and disturbs mitotic fidelity while E6 causes structural chromosomal alterations and DNA breakage (Korzeniewski et al., 2011). The effects on p53 and pRB are considered the molecular signatures of HPV-induced carcinogenesis (Buitrago-Perez et al., 2009). Regression of HPV-induced lesions is mediated by a T-helper 1 lymphocyte (Th1) cell mediated immune response (Stanley, 2012). HPV latency may arise in two ways; (i) low titre infection that is too low to complete the life cycle or (ii) clearance of lesions by the adaptive immune system followed by persistence of low-level viral gene expression, which is reactivated by immunosuppression (Doorbar, 2013).

How HPV initially reaches the conjunctiva is not clear. The prevalence of HPV in OSSN tissue is heterogeneous, varying widely from zero to 100% (Gichuhi et al., 2013).

HPV infection is associated with an increased incidence of HIV acquisition (summary OR = 1.96; 95% CI; 1.55–2.49) (Lissouba et al., 2013).

### 7.2 Failure of DNA repair mechanisms

Several mechanisms prevent UV-induced DNA mutations from being incorporated into the genome and UV-damaged cells from establishing themselves. Cells can correct carcinogen-induced DNA damage; severely damaged cells are eliminated from healthy tissues by processes that trigger apoptosis; and abnormal cells are recognized, targeted and destroyed by immune surveillance (Di Girolamo, 2010).

**Fig. 5:**
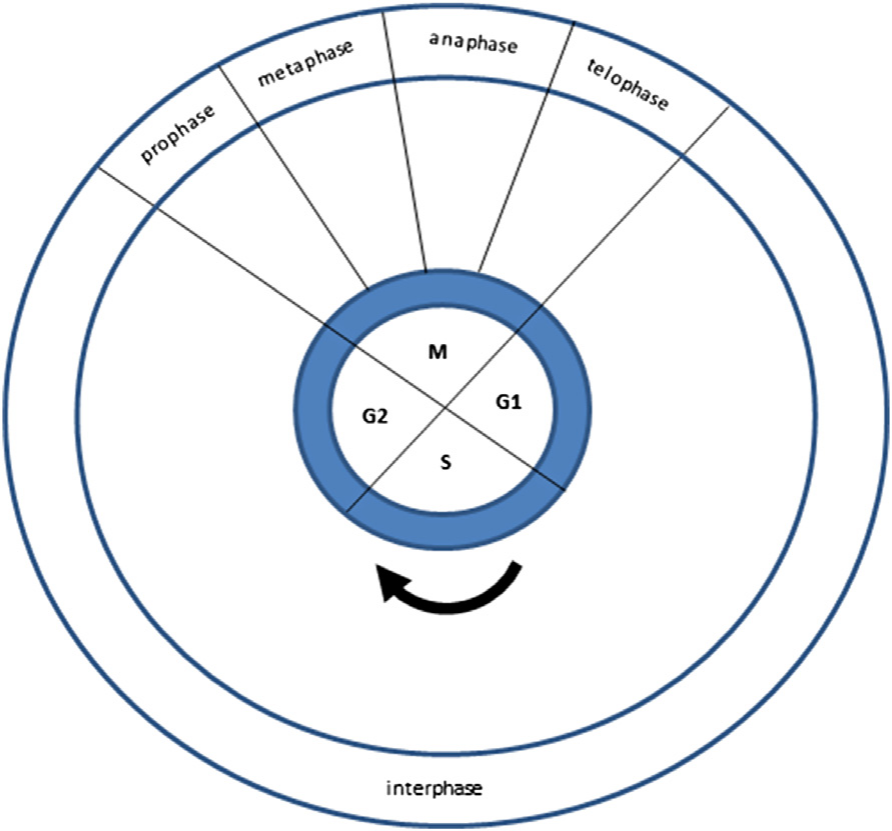
The cell division cycle.

The cell-division cycle involves duplication of the genome and intracellular organelles (Imoto et al., 2011). The stages of the cycle can be visualised directly by high-resolution imaging (Hesse et al., 2012). Nuclear DNA is synthesized during a stage of interphase called the S phase which is followed by a gap (G2), then mitosis (M phase) in which nuclear and cell division occur and another gap (G1) before the next S phase (Fig. 5).

DNA damage activates checkpoint pathways that regulate specific DNA repair mechanisms in the different phases of the cell cycle (Branzei and Foiani, 2008). There are three important cell-cycle checkpoints (Sancar et al., 2004). The G1-S checkpoint prevents cells with damaged DNA from entering the S phase by inhibiting the initiation of replication. The intra-S-phase checkpoint deals with DNA damage that may occur during S-phase or unrepaired damage that escaped the G1-S checkpoint. The G2-M checkpoint prevents cells with damaged DNA from undergoing mitosis.

The molecular mechanisms that repair UVR-induced DNA damage include excision repair, mismatch repair, strand break repair, and cross-link repair (Rastogi et al., 2010). During excision repair sections of damaged DNA are replaced by a nucleotide or base (Sinha and Hader, 2002). The nucleotide excision repair (NER) pathway is primarily responsible for repairing CPDs in humans (Sancar et al., 2004). There are two types of NER; general excision repair which removes lesions from the whole genome and transcription-coupled repair which works on damage in transcribed DNA strands. The latter is not clearly understood but the former is performed through a series of special proteins and proceeds through four discrete steps; recognition of the damage; excision of the section of DNA that includes and surrounds the error; filling in of the resulting gap by DNA polymerase; and sealing of the nick between the newly synthesized and older DNA by DNA ligase (Hu et al., 2013, Reardon and Sancar, 2005).

Base excision repair (BER) is the predominant pathway against lesions caused by ROS, ionizing radiation and strong alkylating agents (Svilar et al., 2011). It proceeds through 5 steps as follows; DNA glycosylases remove damaged or modified bases; the apurimic/apyrimidinic site is removed by an endonuclease or lyase; the remaining phosphate residue is removed by a phosphodiesterase; the gap is filled by a DNA polymerase and the strand sealed by a DNA ligase (Hegde et al., 2008).

#### 7.2.1 The p53 tumour-suppressor system

The p53 tumour-suppressor gene is found on the short arm of chromosome 17 (17p13.1) (Mcbride et al., 1986). P53 is a phosphoprotein found in the nucleus which regulates the cell cycle to protect cells from the effects of DNA damage (Ford, 2005, Jin and Robertson, 2013, Borras et al., 2011). It is thus described as the ‘guardian of the genome’ (Lane, 1992). Once p53 is activated by a stress signal it binds to specific DNA elements in the genome with various primary and secondary response effects (Levine et al., 2006). The primary responses include; cell cycle arrest at the G1-S checkpoint; irreversible withdrawal of cells from the cycle into a terminal state of senescence or programmed cell death (apoptosis) if the damage is irreparable. The secondary responses come from p53-regulated gene products that prevent DNA damage (sestrins) or aid in DNA repair; mediate communication between the cell and its neighbours, the extracellular matrix or more distant cells; or create intracellular or extracellular p53 feedback loops that modulate p53 activity. In addition, deacetylation of p53 facilitates autophagy (autophagocytosis by controlled lysosomal degradation) (Contreras et al., 2013). Mutated p53 (Mutp53) leads to further genomic instability (Meek, 2009).

### 7.3 Reduced immunity

Tumour immunology presumes that tumour cells express antigens such as mutp53 and HPV proteins that distinguish them from non-transformed cells. The immune system prevents tumour formation in 3 ways (i) elimination or suppression of viral infection (ii) preventing establishment of a pro-inflammatory environment and (iii) specifically identifying and eliminating cells that express tumour-specific antigens or molecular signals of cellular stress before they cause harm (Schreiber et al., 2011). The latter is part of a more general process called tumour immuno-editing. Immuno-editing is a dual process in which the immune system may either suppress tumour growth by destroying or inhibiting growth of cancer cells or inadvertently promote tumour progression by selecting tumour cells that are more likely to survive. It has 3 phases: elimination, equilibrium and escape (Schreiber et al., 2011). In the elimination phase the immune system recognizes tumour cells and initiates cell death eliminating them completely before they become clinically apparent. If not fully eliminated the remaining tumour cells enter a state of temporary equilibrium between the immune system and the developing tumour in which the tumour cells remain dormant or continue to accumulate further genetic and epigenetic changes. Finally if the immune system fails to contain the tumour at this phase, surviving tumour cell variants escape causing uncontrolled tumour expansion. The quantity, quality and distribution of tumour infiltrating lymphocytes (TILs) such as CD8+ cytotoxic T lymphocytes (CTL), CD4+ T helper lymphocytes (Th), CD4+ regulatory T lymphocytes (Treg) and CD3+ lymphocytes influence prognosis. A meta-analysis showed that improved survival, measured by the death Hazard ratio (HR), was associated with CD3+ TIL infiltration (HR = 0.58, 95% CI; 0.43–0.78) and CD8+ TIL (HR = 0.71, 95% CI; 0.62–0.82) (Gooden et al., 2011). However TIL counts alone may overestimate this effect and ratios between TIL subsets CD8+/FoxP3+ (effector:regulatory ratio) and CD8+/CD4+ (effector:helper ratio) may be more informative. Natural killer cells are another important component of cancer immunosurveillance and immuno-editing (Gross et al., 2013).

#### 7.3.1 Photoimmunosuppression

Ambient UV radiation suppresses cell-mediated immunity (Clydesdale et al., 2001). UVB is a more potent immunosuppressor than UVA (Poon et al., 2005). This phenomenon referred to as photoimmunosuppression is not limited to exposed cutaneous tissues but is also systemic, affecting internal organs (Gibbs and Norval, 2013). The immunosuppressive effect of UVB is used in phototherapy of skin conditions such as psoriasis (Chen et al., 2013). In the skin, UVB stimulates migration of epidermal Langerhans cells, which present antigens to lymphocytes in the draining lymph nodes promoting a Th2 and regulatory T cells (Treg) dominated response that suppresses local immune responses (Taguchi et al., 2013, Schwarz and Schwarz, 2011, Norval et al., 2008).

#### 7.3.2 HIV

HIV preferentially infects helper T cells (CD4+), inducing their apoptosis (Lundin et al., 1987, Cloyd et al., 2001, Alimonti et al., 2003). The virus establishes latency in resting memory T cells, which explains why combination antiretroviral therapy (ART) is not curative; interruption of treatment inevitably results in rebound viraemia (Van Lint et al., 2013) (van Der Sluis et al., 2013). HIV may weaken tumour immunosurveillance (Mbulaiteye et al., 2003). A meta-analysis found an increased incidence of cancers among both HIV/AIDS patients and immunosuppressed transplant recipients, however, there was no significant difference in the risk between the two groups suggesting that it is primarily the immunosuppression, rather than another action of HIV that is responsible for the increased risk of cancer (Grulich et al., 2007).

HIV potentiates the oncogenic action of other viruses such as HPV, Kaposi sarcoma-associated herpes virus (KSHV) and Epstein–Barr virus (EBV) by enhancing their transmission to target cells (Aoki and Tosato, 2004). A report from Botswana reported multiple oncogenic viruses (EBV, HPV, KSHV, HSV1/2 and CMV) in cases of OSSN and pterygium (Simbiri et al., 2010). In rabbits immunosuppression induced by T-cell depletion facilitated reactivation of latent HPV infection leading to a 3 to 5 log increase in the number of viral copies to levels associated with productive infection (Maglennon et al., 2014).

Lastly, HIV induces a state of persistent inflammation (Hunt, 2012). Markers of inflammation such as C-reactive protein and interleukin-6 are elevated in HIV patients particularly those on ART even when viral loads are undetectable (Neuhaus et al., 2010). Inflammatory microenvironments have tumour-promoting effects (Mantovani et al., 2008). One proposed mechanism is via overexpression of microRNA-155 (MiR155), which increases spontaneous mutation rates (Tili et al., 2011, Valeri et al., 2010).

#### 7.3.3 Vitamin A deficiency

Vitamin A helps to maintain the integrity of the ocular surface (Kanazawa et al., 2002). Its deficiency is associated with squamous metaplasia of the conjunctiva (Mckelvie, 2003). Vitamin A also acts as a mucosal and systemic immune enhancer through immuno-homeostasis of CD4+ helper T cells and Treg cells (Hall et al., 2011, Pino-Lagos et al., 2011, Ross, 2012). These cells are part of tumour immuno-surveillance. Retinoids are reported to prevent various cancers in the skin and liver (Alizadeh et al., 2014). They promote stem cell differentiation through epigenetic modifications of histones or by altering chromatin structure to remove the stem cell from the self-renewing pluripotential state to a differentiated one (Gudas, 2013). Loss of the differentiated phenotype can lead to generation of cancer stem cells (Gudas, 2013). Retinoids activate DNA transcription in stem cells via retinoic acid receptors (RAR α,β,γ), retinoid X receptors (RXR α,β,γ) and other transcription factor regulatory proteins (Gudas and Wagner, 2011).

A study in Kenya found that HIV-positive women had a higher prevalence of vitamin A deficiency (<30 μg/dL) than HIV-negative women (59% vs 29%, *p* < 0.001) (Baeten et al., 2002).

We hypothesize that vitamin A deficiency has three effects; it compromises the integrity of the surface epithelium creating micro-abrasions for HPV entry, it leads to cell-mediated immunodeficiency, and dysregulation of stem cell differentiation.

## 8 Downstream events after initiation of neoplasia

### 8.1 Uncontrolled cell replication

Human somatic cells can only undergo a limited number of cell divisions (50–70) then arrest, an event related to shortening of telomeres (Gomez et al., 2012). In comparison, epidermal stem cells can divide for more than 150 generations in vitro (Mathor et al., 1996). Telomeres are a repetitive sequence of nucleotides rich in guanidine, synthesized by the enzyme telomerase, that cap the ends of chromosomes to prevent chromosomes from deterioration (Blackburn and Gall, 1978). Usually telomeres shorten during each round of DNA replication but in advanced cancers telomerase is reactivated to maintain telomere length allowing many more cell divisions (Artandi and Depinho, 2010, Prescott et al., 2012). Downregulation of 14-3-3σ protein in keratinocytes maintains telomerase activity allowing them to escape replicative senescence (Dellambra et al., 2000).

### 8.2 Angiogenesis

Neovascularization occurs to meet the increased tumour metabolic demand. Tumours overproduce vascular growth factors such as vascular endothelial growth factor (VEGF) (Aonuma et al., 1999). In conjunctival tumours this manifests clinically as feeder vessels, enlarged blood vessels in the conjunctiva that perfuse the growth. In a Tanzanian study 88% of OSSN lesions and 61% of benign tumours had feeder vessels (Nguena et al., 2014).

### 8.3 Metastasis

Metastasis is considered a hallmark of malignancy. Cells loose adherence with each other and secrete proteolytic enzymes such as matrix metalloproteinases (MMPs) that degrade the extracellular matrix (Chiang and Massague, 2008). UVB radiation alters the balance between MMPs and tissue inhibitors of matrix metalloproteinases (TIMPs) (Ng et al., 2008). When exposed to UVB, cultured human dysplastic conjunctival epithelial cells show increased expression of MMP-1 and MMP-3 with little change in TIMP-1 unlike normal conjunctival cells (Ng et al., 2008). Increased MMP activity upsets cell-to-cell adhesion and promotes carcinogenesis and tumour invasion into surrounding tissues (Johansson et al., 2000). In lesions of squamous cell carcinoma of the conjunctiva MMPs and TIMP are overexpressed compared to normal conjunctiva and cornea (Di Girolamo et al., 2013).

## 9 Are cancer stem cells central to OSSN?

The short lifespan of ocular surface cells means that epithelial cells with DNA mutations do not last long enough to have an effect, as they are constantly being shed from the surface. The longevity of stem cells however gives them enough time to accumulate mutagenic insults. Why tumours are heterogeneous yet originating from the same stem cells could partly be explained by ongoing mutagenesis (Pardal et al., 2003).

The location of stem cells on the ocular surface coincides with the position of OSSN tumours while the growth pattern of lesions (from base upwards) is consistent with the stem cell theory. A study in Australia described concurrent existence of features of OSSN and primary acquired melanosis and stem cells arranged in micro-clusters in the basal epithelium in 12% of pterygiums (Chui et al., 2011). Focus would necessarily have to shift to ocular surface limbal epithelial stem cells (LESCs) as the potential progenitors of OSSN to consider new explanations for tumour formation, new diagnostic methods to detect the LESCs and new treatments that target LESCs.

## 10 Conclusion

The known risk factors of OSSN – solar UV radiation, HIV and HPV are implicated in the aetiology. The pattern of distribution of OSSN lesions within the interpalpebral fissure of the ocular surface at the limbus, particularly the nasal side provides further clues. The site is highly vulnerable to solar UVR and has a high concentration of stem cells in the basal epithelium. Stem cells in the limbal epithelial crypts are the likely originators of this disease, and may take on cancer stem cell properties.

Neoplasia is probably initiated when background solar UV radiation causes various forms of genetic and epigenetic DNA damage. UVB mainly creates pyrimidine dimers. Specific dimer CC → TT transformation, a signature UV mutation, occurs at the p53 gene, a tumour suppressor that maintains cell-cycle arrest at the G1-S checkpoint. UV radiation also reactivates latent viruses such as HPV. HPV's E7 protein keeps infected cells in a proliferative state while E6 inhibits cell cycle arrest of DNA-damaged cells. Immunosuppression caused by UV radiation, HIV and vitamin A deficiency weakens the tumour surveillance system and allows DNA-damaged cells to proliferate into tumours. Vitamin A deficiency interferes with ocular surface integrity creating micro-abrasions through which HPV may invade the conjunctival basement membrane and epithelial cells. Cancer cells reactivate telomerase which maintains long telomeres increasing the number of cell divisions a cell can undergo. Further tumour expansion and metastasis is enhanced by angiogenesis and increased matrix metalloproteinases (MMPs) which destroy the intercellular matrix.

Despite these advances in our understanding there remain gaps, which are areas for further research. For example, there is no explanation why the disease is mostly unilateral despite both eyes receiving equal sunlight exposure. Equally the route of transmission of HPV to the conjunctiva is unknown. The drivers on a molecular level which convert intraepithelial neoplasia to squamous cell cancer are also out with our current understanding of the disease.

## Figures and Tables

Reprinted by permission from Macmillan Publishers Ltd: Eye 20 (8):893–899 copyright 2006

**Table 1:** Molecular markers for limbal stem cells (Schlotzer-Schrehardt and Kruse, 2005).

Characterization	Examples
Cytoskeletal proteins - proteins that form intermediate filaments in epithelial cells	i) Cytokeratins^a^ (CK3, CK12, CK5, CK14, CK19) – CK3/CK12 pair is lacking in the limbus. It is a marker of corneal phenotype ii) Vimentin fastens limbal stem cells to their local environment. It is localised in limbal basal cells
Cytosolic proteins - associated with cellular metabolic functions	a) enzymes i) cytochrome oxidase – Na/K-ATPase ii) carbonic anhydrase iii) α-enolase (initially thought to be a glycolytic enzyme it acts as a plasminogen binding receptor) iv) protein kinase C (PKC), a key enzyme controlling signal transduction pathways in growth and differentiation. v) aldehyde dehydrogenase (ALDH) vi) transketolase (TKT) b) Cell-cycle associated proteins i) Cyclins ii) Ki67 acts as a marker for actively cycling cells c) Metallothioneins, which are cysteine-rich metal-binding intracellular proteins d) Involucrin, a structural protein found in the cytosol of differentiated human keratinocytes e) calcium-linked protein (CLED), that is associated with early epithelial differentiation f) protein S100A12, which is involved in Ca^2 +^-dependent signal transduction processes in differentiated cells
Nuclear proteins	p63 is a transcription factor that regulates epithelial development and differentiation. Although concentrated at the limbus in stem cells, it is not exclusively expressed by stem cells.
Cell surface proteins a) Cell–cell and cell–matrix interaction molecules b) Growth factor receptors c) Transporter molecules d) Cell surface glycoconjugates	i) Connexins are transmembrane proteins in gap junctions that allow diffusion of ions, low molecular weight metabolites, and second messengers thus determining the extent of metabolic cooperation between cells ii) Cadherins are a family of Ca^2+^-dependent transmembrane receptors that mediate cell–cell adhesion iii) Integrins are a large family of heterodimeric transmembrane glycoproteins consisting of α and β subunits, that play a role in attachment of cells to the basement membrane, extracellular matrix proteins or to ligands on other cells iv) epidermal growth factor receptor (EGF-R) v) keratinocyte growth factor receptor (KGF-R) vi) TrkA, the high affinity receptor for nerve growth factor (NGF). vii) hepatocyte growth factor (HGF) viii) Transferrin receptor CD71 ix) transforming growth factor-beta (TGF-β) type I and II x) ABCG2, a member of the ATP binding cassette transporters. ABCG2 has been proposed as a universal and conserved marker for stem cells from a wide variety of tissues. ABCG2 protein is also known as breast cancer resistant protein 1 (BCRP1), which causes resistance to certain chemotherapeutic drugs. It is a multi-resistance drug protein that pumps drugs out of cells. This is protective to the cell. It is localized to the cell membrane and cytoplasm of some human limbal basal epithelial cells, but not in most limbal suprabasal cells and corneal epithelial cells. xi) α-2,3-sialyltransferase
Neuronal markers - human corneal and limbal cells may exhibit neuronal properties characteristic of their neuroectodermal origin	i) Nestin is a neural stem cell marker. It is not normally expressed in limbal basal cells except when they are in an environment with mitogens ii) transcription factor Pax-6
Hematopoietic stem cell markers	i) CD34 a sialomucin cell surface antigen ii) CD133 a transmembrane glycoprotein

a In the original article cytokeratins were abbreviated as K. We have modified that to CK in keeping with more recent terminology.
